# The Initial Learning and Supply Cost Curve of Incorporating Interlaminar and Transforaminal Endoscopy at a Tertiary Academic Medical Center

**DOI:** 10.7759/cureus.102503

**Published:** 2026-01-28

**Authors:** Mark M Zaki, Edward S Harake, Varun G Kathawate, Michael J Strong, Rushikesh S Joshi, Joseph R Linzey, Yamaan S Saadeh, Osama N Kashlan

**Affiliations:** 1 Neurosurgery, University of Michigan, Ann Arbor, USA; 2 Neurosurgery, University of Pennsylvania, Philadelphia, USA; 3 Neurosurgery, Columbia University, New York, USA; 4 Neurological Surgery, Weill Cornell Medicine, New York, USA

**Keywords:** interlaminar, learning curve, minimally invasive spine surgery, retrospective review, transforaminal endoscopy

## Abstract

Introduction

Spinal endoscopy is challenging with a steep learning curve and an unclear supply cost curve. We describe the initial learning and supply cost curves for a surgeon who incorporated interlaminar and transforaminal endoscopy.

Methods

Interlaminar and transforaminal cases performed between November 2021 and May 2023 were retrospectively reviewed. Linear regressions were used to assess the effect of experience on operative time, intraoperative X-ray utilization, and surgical supply costs. Trends were assessed using a cumulative running median analysis to determine a cutoff point for surgeon familiarity.

Results

A total of 56 patients were included (41 interlaminar and 15 transforaminal). Surgical time per decompressed level was similar (192 minutes ± 60.9 vs. 213 minutes ± 80,* P* = 0.3). Transforaminal cases had greater X-ray utilization (42.7 images ± 23.5 vs. 105.4 images ± 63.3, *P* < 0.01) and supply cost ($1,223 ± 389 vs. $2,090 ± 978, *P* < 0.01). Cutoff points for familiarity were earlier in the interlaminar group in X-ray use (nine cases vs. 11) and supply cost (two cases vs. 11). Both approaches had the same operative time cutoff in three cases. Differences did not result in significantly different surgical complications, disposition, or revision rates.

Conclusion

The learning curve for spinal endoscopy is non-negligible. The supply cost per decompressed level and X-ray utilization were cheaper for the interlaminar approach. Time per decompressed level appeared similar. Experience led to significantly reduced intraoperative X-ray utilization, a trend towards reduced intraoperative time, and stable supply costs. Cutoff points to familiarity were earlier in the interlaminar group, but surgical outcomes were similar pre- and post-cutoff.

## Introduction

Surgical decompression effectively relieves spinal cord and nerve root compression, leading to improvement in radiculopathy, myelopathy, and neurogenic claudication [1,2]. Despite demonstrated efficacy with traditional open decompression, these procedures require extensive manipulation and dissection of paraspinal musculature and disruption of the posterior ligamentous tension band. Minimally invasive spine surgery (MISS) techniques were developed to preserve adequate treatment while minimizing anatomic disruption to surrounding structures [3-5]. MISS techniques offer decreases in blood loss, operative duration, hospital stay, and recovery time [6-9].

MISS is performed microscopically or endoscopically. Microscopic decompression uses a microscope for anatomic visualization and is performed via a soft tissue tubular retractor. Despite the benefits relative to open surgery, the tubular technique is limited by a small field of view and a rigid working channel [10,11]. Endoscopic spine surgery has seen growing popularity given reductions in tissue disruption, blood loss, and postoperative back pain [10-14]. Furthermore, constant irrigation in endoscopy dramatically reduces the risk of infection [15]. Water pressure dissects the planes and pushes the dura away, which may reduce the risk of cerebrospinal fluid (CSF) leak [16]. The adoption of endoscopic spine surgery has been supported by the technological evolution of endoscope models [17,18] and the expansion of surgical indications from disc herniation [14,19] to degenerative stenosis [20]. There are many options within the endoscopic arsenal to selectively intervene on these spinal pathologies using uniportal or biportal approaches within the interlaminar or transforaminal window [14,18,19,21].

While its applications are vast, endoscopic surgery remains a technologically challenging procedure to learn [22]. Furthermore, the transforaminal approach is not a common view utilized by spine surgeons [23]. Setup, unfamiliar equipment, and view thus pose a potentially steep learning curve for interested spine surgeons [24,25]. There is a paucity of data, however, that characterizes the learning curve for endoscopic spine surgery, especially at an academic center where attendings, fellows, and residents each have their own learning curves that affect the total learning curve.

We thus sought to explore the initial learning and supply cost curve of a spine surgeon who incorporated interlaminar and transforaminal endoscopy at a tertiary academic medical center.

## Materials and methods

Study design and outcomes

This study received approval from the University of Michigan Institutional Review Board (approval number: HUM00209228), which granted a waiver of informed consent. We conducted a retrospective review of all interlaminar and transforaminal cases from the first case on November 16, 2021, to May 5, 2023. All cases were performed by the same attending surgeon at a tertiary academic medical center. Patients were included in this study if they underwent an endoscopic spinal decompression using a uniportal interlaminar or transforaminal approach. Patients were otherwise excluded if their surgery was not performed using endoscopy or was part of a multi-approach planned operation.

Demographic information was collected, including age at presentation, sex, and body mass index (BMI). Risk factors that could impact bleeding and wound healing (i.e., diabetes, bleeding disorders, and anticoagulation) were also included to further characterize the patient population. The following surgical information was recorded: diagnosis/surgical indication, prior spine surgical history, interlaminar vs. transforaminal approach, number of levels operated on, total operation time, number of intraoperative X-ray images obtained, estimated blood loss (EBL), total cost of intraoperative supplies, and discharge destination.

The primary outcomes of interest for this study were per-level operative time, the number of X-ray images obtained, and supply cost as a function of the number of weeks from the first case and the total number of cases for the operating surgeon. While the total case number was used to assess the change in these intraoperative variables with greater approach-based experience, weeks were used to account for the impact of surgical frequency. Supply-cost was defined as the total monetary cost of materials used during surgery, including electrosurgical probes, medications, and other disposable supplies (e.g., drapes and sutures). This was obtained from the postprocedural internal accounting log of all used supplies and their summative cost.

A regression line was fitted for each outcome based on the surgical approach (interlaminar vs. transforaminal) to highlight the relationship of the respective metric with increased experience. Prior to regression analyses, outlier points within each outcome were filtered out to reduce their impact on the best-fit line. Additionally, a plot with the cumulative running median of each outcome was used to visualize the per-metric rate and direction of change. The running median was used as opposed to the running average to account for possible outlier values. A cutoff point of familiarity was determined where the pre- and post-cutoff point averages of all points most significantly differed. We enforced that the pre-cutoff point must have a higher value than the post-cutoff point to specifically capture points of relative improvement. This cutoff point was used to reflect the point at which the team reached maximal surgical proficiency. Demographic information and comorbidities were subsequently recorded at pre- and post-cutoff times in both approaches to account for potential confounders other than the primary outcomes.

The secondary outcomes of interest spanned both surgical safety and clinical outcomes. Safety variables included EBL per level over the same time scales, along with rates of durotomy. Reported surgical outcomes included rates of revision surgery as well as changes in patient-reported outcomes measured via the validated Visual Analogue Scale (VAS). VAS measures pain on a scale of 0 to 100, from "no pain" to "worst pain." In this study, postoperative VAS was obtained from follow-up visits within six months of surgery. These outcomes were reported and summarized at both pre- and post-cutoff points to evaluate significant changes in surgical outcomes following proficiency. EBL was measured by subtracting the amount of irrigation utilized from the total volume collected in the suction canister. Durotomies were assessed via intraoperative visual inspection of dural tearing and via postoperative assessment of CSF leak symptoms (e.g., postural headache).

Surgical techniques

**Figure 1:**
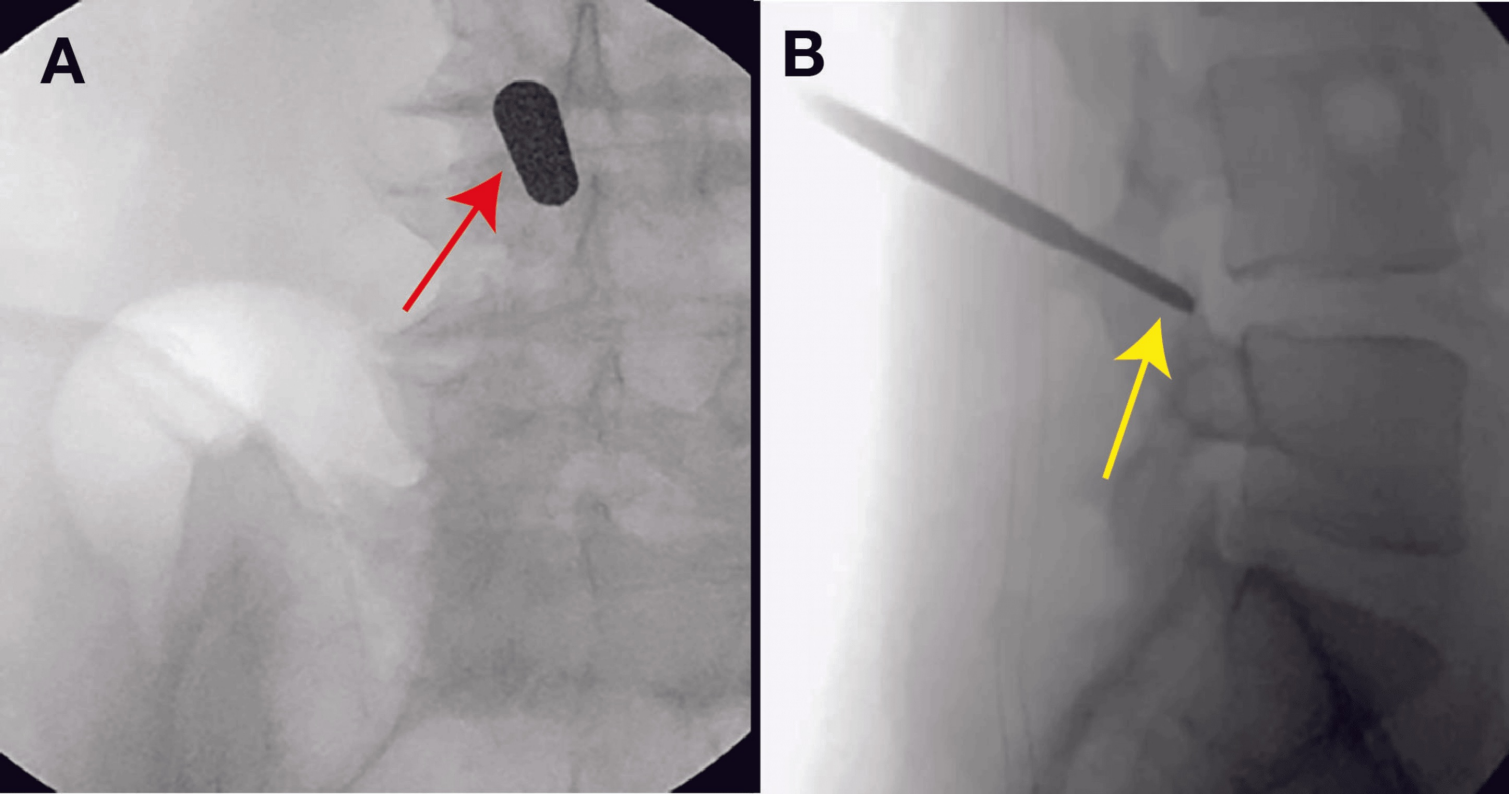
Intraoperative Fluoroscopic Images Demonstrating Endoscopic Decompression Approaches Intraoperative fluoroscopic snapshots from endoscopic decompressions using (A) an interlaminar and (B) a transforaminal approach. A dilator (red arrow) and Jamshidi needle (yellow arrow) are shown, which were used to access the surgical site and facilitate placement of the endoscope in the interlaminar and transforaminal approaches, respectively.

All patients were sedated with general anesthesia and positioned prone with pressure points padded. Fluoroscopy was utilized to plan the trajectory for precise vertebral level access before making a small incision. Subsequent steps were predicated upon the planned procedural approach. In interlaminar laminectomies (Figure 1A), serial dilators were used to dissect down to the bone. A high-speed drill was used for minimally invasive paraspinal muscular dissection down to bone, and a high-speed drill was used to perform the laminectomy. A combination of Kerrison rongeurs and graspers was used for resection of the ligamentum flavum for optimal visualization of the thecal sac and nerve roots before achieving bilateral decompression. In transforaminal microdiscectomies (Figure 1B), superior articulating process docking was obtained using a Jamshidi needle, and reamers were subsequently used to expand the foramen and facilitate the working channel. After placing a working tube and scope, the compressed nerve root was visualized before dissecting disc fragments and the ventral epidural space. A flexible grasper, high-speed drills, and Kerrison rongeurs were used to optimize the decompression.

Data analysis

Patient data were collected from the electronic health record, and supply cost information was obtained from hospital charge data. Categorical data were compared using chi-squared analysis; continuous data were compared with a two-sample t-test. Outlier filtering was done by finding the points 1.5 * interquartile range (IQR) above quartile 3 and below quartile 1 of the data. Data analysis and statistical hypothesis testing were conducted using Microsoft Excel (Microsoft Corporation, Redmond, Washington) and Python programming on Google Colab/Jupyter Notebook. Linear regression functionality from both tools was used to determine per-level operative time, X-ray use, and supply cost over time/case number.

## Results

A total of 56 endoscopic spine surgical cases were identified from November 16, 2021, to May 5, 2023, of which 41 were performed using an interlaminar approach and 15 were performed using a transforaminal approach (Table 1). To account for the disproportionate sizes of the two groups, demographic and surgical characteristics were reported for both the whole interlaminar group and the first 15 cases for a size-matched comparison with the transforaminal approach.

**Table 1:** Patient Demographics * Indicate a statistically significant difference between the two patient subgroups. Abbreviations: BMI, body mass index; N/A, not applicable.

Characteristic	Overall	Interlaminar	Interlaminar: First 15 Cases	Transforaminal	P-value: All Cases	P-value: First 15 Cases
No.	56	41	15	15	N/A	N/A
Age (years)	59.8 ± 18.3	64.3 ± 17.0	60.6 ± 19.0	47.7 ±16.3	0.002*	0.055
Sex						
Male	29	21	8	8	0.89	1
Female	27	20	7	7	N/A	N/A
BMI	30.3 ± 6.4	30.1 ± 7.0	31.4 ± 8.1	30.9 ± 4.7	0.68	0.84
Diabetes	9	7	1	2	0.74	0.54
Bleeding disorder	4	4	2	0	0.21	0.14
Anticoagulation	4	4	0	0	0.21	1
Prior spine surgery	11	8	2	3	0.97	0.62

Demographics

The average (± SD) participant age was 59.8 (± 18.3) years, with 27 female patients (48.1%) in total. The average patient age of all interlaminar cases was significantly higher than that of transforaminal cases (64.3 ± 17.0 vs. 47.7 ± 16.3, *P* = 0.002). However, this difference was not observed when comparing the sample size-matched groups (60.6 ± 19.0 vs. 47.7 ± 16.3; *P* = 0.055). There were no other significant differences between groups in patient demographics or comorbidities, including sex, BMI, diabetes, bleeding disorders, anticoagulation use, or prior spine surgery.

Surgery-specific characteristics

Significant differences in preoperative symptomatology and imaging findings were observed between the interlaminar and transforaminal groups (Table 2). There was a significantly higher rate of neurogenic claudication among patients in the interlaminar group (32 cases vs. one case; *P* < 0.001), but both groups had similar rates of myelopathic (*P* = 0.3) and radicular symptoms (*P* = 0.53). Regarding imaging findings, lumbar stenosis in the interlaminar group was more likely to be associated with ligamentum flavum hypertrophy (26 cases vs. one case; *P* < 0.001) and spondylolisthesis (11 cases vs. 0; *P* = 0.03), while the transforaminal group had greater evidence of disc herniation (three cases vs. 14; *P* < 0.001). Transforaminal cases had a significantly greater proportion of cases with compression in the thoracic region (four cases vs. 0; *P* < 0.001). When comparing the size-matched cohorts, all differences remained significant except for a higher but nonsignificant rate of spondylolisthesis in the interlaminar group (Table 2).

**Table 2:** Surgery-Specific Characteristics * Indicate a statistically significant difference between the two patient subgroups. Abbreviations: EBL, estimated blood loss; IL, interlaminar; IPR, inpatient rehabilitation; SAR, subacute rehabilitation; TF, transforaminal; VAS, visual analogue scale.

Characteristics	Overall	Interlaminar	Interlaminar: (First 15 Cases)	Transforaminal	P-value: All Cases	P-value: First 15 Cases
Clinical symptoms						
Neurogenic claudication	33	32	9	1	1.5E-06*	0.002*
Myelopathy	7	4	3	3	0.30	1
Radiculopathy	26	18	10	8	0.53	0.46
Imaging findings						
Ligamentum flavum - hypertrophy	27	26	8	1	7.80E-05*	0.005*
Spondylolisthesis	11	11	3	0	0.03*	0.07
Disc herniation	17	3	3	14	5.70E-10*	5.1E-05*
Area of compression						
Cervical	4	4	3	0	0.21	0.07
Thoracic	4	0	0	4	6.00E-04*	0.03*
Lumbar	49	37	12	12	0.30	1
No. of decompressed levels	1.2 ± 0.4	1.3 +/- 0.5	1.3 +/- 0.5	1 ± 0	0.02*	0.03*
Operative time (min)/level	197.8 ± 66.5	192.1 ± 60.9	194.4 ± 55.3	213.3 ± 80.3	0.30	0.46
No. X-ray images/level	57.8 +/- 45.3	42.7 ± 23.5	57.7 ± 30.9	105.4 ±63.3	1.90E-06*	0.02*
Supply cost/level	1455.5 ± 709.9	1223.3 ± 389.1	1214.8 ± 435.4	2090.2 ± 978.5	1.40E-05*	0.004*
EBL (cc)/level	9.1 ± 7.8	9.0 ± 8.7	6.8 ± 3.7	9.4 ± 5.2	0.86	0.13
Discharge						
Home	52	38	14	14	0.93	1
SAR/IPR	2	1	1	1	0.45	1
Nursing home	2	2	0	0	0.38	1

**Figure 2:**
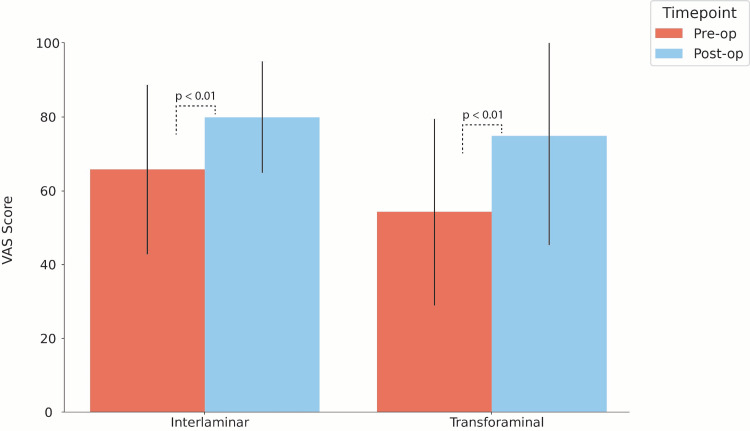
Pre- and Postoperative VAS Scores by Endoscopic Approach Paired bar plots exhibiting pre-/postoperative patient-reported outcomes based on the endoscopic approach. Each bar demonstrates the mean Visual Analogue Scale (VAS) with associated standard deviation lines. *P-*values are derived from paired *t*-tests.

Intraoperatively, interlaminar cases were performed as one- or two-level decompressions, while all transforaminal discectomies were one level. Transforaminal cases, on average, utilized more resources per vertebral level than interlaminar cases, including the number of X-rays obtained/level (105.4 ± 63.3 images vs. 42.7 ± 23.5 images; *P* < 0.001) and supply cost/level ($2,090 ± 978.5 vs. $1,223 ± 389.1; *P* < 0.001). This remained significant when controlling for sample size, both for X-ray acquisition rates (105.4 ± 63.3 images vs. 57.5 ± 30.9 images; *P* = 0.02) and supply cost ($2,090 ± 978.5 vs. $1,215 ± 435.4; *P* < 0.01). While the average operative time/level was also greater in transforaminal procedures, this difference was not statistically significant. Ultimately, differences in resource utilization did not result in different intraoperative complications or disposition, as both groups had similar rates of EBL/level and discharge locations. Following surgery, patients exhibited significantly improved VAS (*P* < 0.001) in both the interlaminar (n = 15; +35.1 ± 59.8%) and transforaminal approaches (n = 6; +43.1 ± 37%) (Figure 2).

Regression analysis

**Figure 3:**
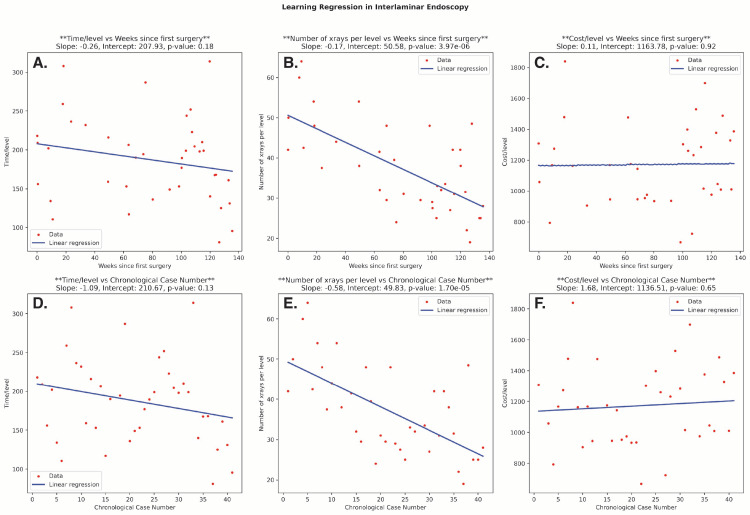
Linear Regression Analyses of Surgical Outcomes From an Interlaminar Approach Plots depicting linear regression analyses for the following surgery-specific outcomes from an interlaminar approach: operative time/instrumented level, X-ray images obtained/instrumented level, and supply cost/instrumented level. A–C: The relationship of each outcome to weeks from the first case is evaluated. D–F: The same outcomes are assessed for their relationship to increasing case number. Note: The formula for each best-fit regression line is presented in the top right corner of each subplot.

Regression analysis was conducted in both the interlaminar and transforaminal groups to assess the relationship between resource use and time (in weeks) and case number. Among interlaminar cases, there was a decrease in operative time/level and X-rays/level over time and case number. While this was not significant in operative time (weeks: P = 0.18; case number: P = 0.13), the relationship was significant in X-ray usage across the number of weeks (P = 3.97e-06) and absolute case number (P = 1.70e-05) (Figure 3).

**Figure 4:**
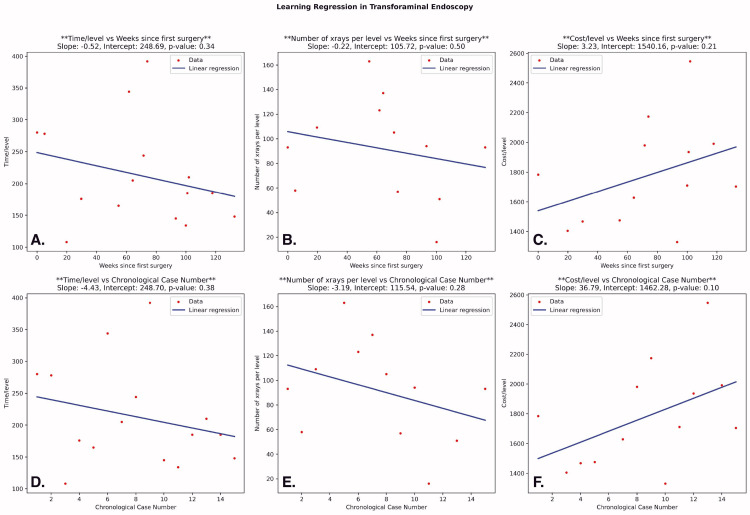
Linear Regression Analyses Following a Transforaminal Approach Linear regression analyses for operative time/instrumented level, X-ray images obtained/instrumented level, and supply-cost/instrumented level following a transforaminal approach. A–C: Each subplot evaluates the relationship of the corresponding surgical outcome to weeks from the first case. D–F: The relationship of the surgical outcome is assessed with respect to increasing case number. Note: The formula for the best-fit regression line is presented in the top right corner of each subplot.

There was a slight increase in supply cost/level in the interlaminar cases; however, this was not significant across either week number (P = 0.92) or case number (P = 0.65). In transforaminal cases, there was a decrease in usage of all resources over time and case number, with a steeper decline in X-ray usage. However, these trends were not statistically significant (Figure 4).

Cumulative running median analysis

**Figure 5:**
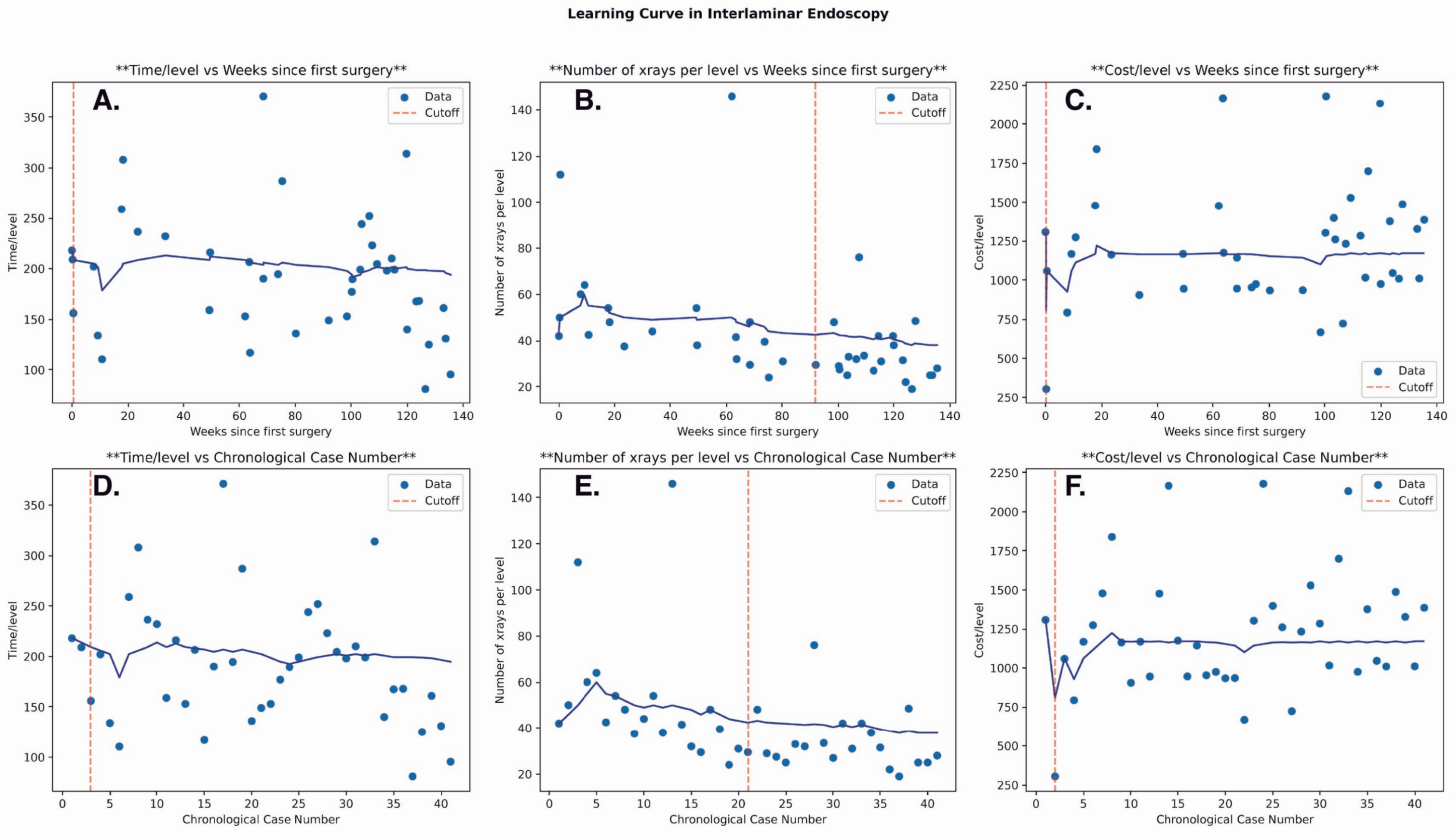
Cumulative Running Median for Surgical Outcomes Following Both Interlaminar and Transforaminal Approaches A cumulative running median analysis for each surgical outcome relative to weeks from the first case (A–C) and increasing case number (D–F) following an interlaminar approach. Compared to linear regression, the cumulative running median is useful for assessing the dynamic trend of each outcome's change with greater experience and for determining a cutoff point at which procedural familiarity is achieved. The cutoff point of familiarity is defined as the point of greatest statistically significant difference in outcome average between two given weeks (A–C) or cases (D–F).

Using cumulative running median analysis to assess general trends, a cutoff point of familiarity was determined among all resource types in both groups. In the interlaminar group, the overall cutoff point of competency as measured by operative time/level and X-ray/level was found at 0.5 weeks/case 3 (P = 0.002) and 92 weeks/case 21 (P = 1.2e-08), respectively. While not significant, the cutoff point based on supply cost/level was the earliest at 0.25 weeks/case 2 (P = 0.13) (Figure 5).

**Figure 6:**
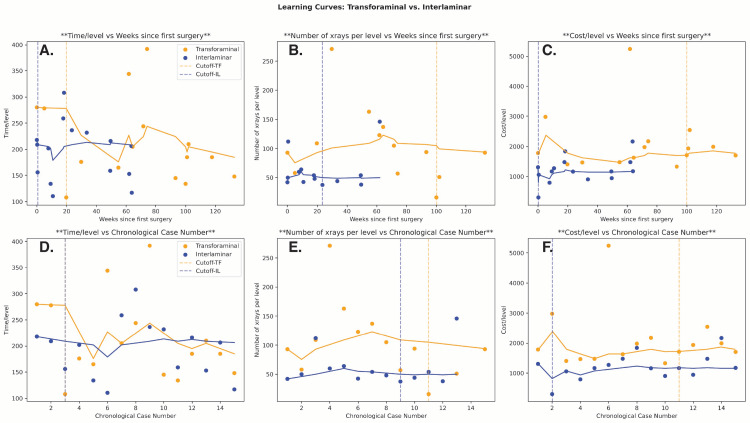
Cumulative Running Median for Surgical Outcomes Following Both Interlaminar and Transforaminal Approaches The median values for each outcome are followed by greater experience measured by weeks following the first case (A–C) and increasing case number (D–F). To facilitate more direct comparison, the first 13-15 cases of the interlaminar approach are included to match the transforaminal sample size based on outcome. The cutoff point of familiarity is defined as the point of most significant difference in median outcome value between two given weeks or cases.

Within the transforaminal group, cutoff points in operative time/level were found at 19.75 weeks/case 3 (P = 3.5e-06). Cutoffs were the same for X-ray utilization and supply cost at 100 weeks/case 11 (P = 0.5 and 0.86, respectively) (Figure 6). Notably, cutoffs for X-ray usage in transforaminal cases were based on 13 cases since two cases did not have available data. Given the relatively small transforaminal sample size compared to the interlaminar group, cutoff points in the first 13 to 15 interlaminar cases were assessed to draw a more direct comparison. Among operative time, X-ray use, and supply cost, cutoffs were found at 0.5 weeks/case 3 (P = 0.01), 23.5 weeks/case 9 (P = 0.63), and 0.25 weeks/case 2 (P = 0.1). These initial interlaminar cases were all completed within 68.5 weeks, while the transforaminal cases as a whole were completed within 132.75 weeks.

We compared patient demographics, comorbidities, and outcomes pre- and post-cutoff points for competency based on operative time/level. In the interlaminar group, patient characteristics, intraoperative complications, and revision surgery rates were similar between the pre- (n = 3 cases) and post-cutoff (n = 38 cases) groups. In the transforaminal group, data remained similar between the pre- (n = 3 cases) and post-cutoff (n = 12 cases) groups (Tables 3, 4). In both approaches, there was a slightly higher rate of radiculopathy in the post-cutoff group (Table 3; *P* = 0.04). Furthermore, all intraoperatively identified durotomies were characterized as small dural tears and sealed with a collagen- or fibrin-based sealant. There were no instances of symptomatic CSF leak.

**Table 3:** Demographics Pre- and Postoperative Time Cutoff Abbreviations: BMI, body mass index; EBL, estimated blood loss; N/A, not applicable; No., number.

Demographics	Interlaminar			Transforaminal		
	Pre-cutoff	Post-cutoff	P-value	Pre-cutoff	Post-cutoff	P*-*value
No.	n = 3	n = 38	N/A	n = 3	n = 12	N/A
Age (years)	60.3 ± 23.1	64.9 ± 16.4	0.6	61 ± 14.2	45.1 ± 16	0.1
Sex						
Male	3	18	0.08	1	7	0.44
Female	0	20	N/A	2	6	N/A
BMI (kg/m^2^)	25.5 ± 2.5	30.4 ± 7.2	0.18	30.4 ±7.0	31.7 ± 4.5	0.66
Diabetes	0	7	0.41	0	2	0.45
Bleeding disorder	0	4	0.55	0	0	N/A
Anticoagulation	0	4	0.55	0	0	N/A
Prior spine surgery	0	8	0.38	1	2	0.52
Surgical outcomes EBL/level	4.6 ± 3.9	9.5 ± 8.9	0.29	6.25 ± 2.5	10.1 ± 5.5	0.21
Durotomy	0	6	0.4	0	1	0.6
Revision surgery	0	5	0.5	0	0	N/A

**Table 4:** Operative Following Operative-Time Familiarity Cutoff Abbreviations: CSF, cerebrospinal fluid; EBL, estimated blood loss.

Outcomes	Interlaminar			Transforaminal		
	Pre-cutoff	Post-cutoff	P-value	Pre-cutoff	Post-cutoff	P-value
Number	n = 33	n = 8	N/A	n = 9	n = 6	N/A
EBL/level	9.3 ± 9.2	7.0 ± 2.4	0.59	8.3 ± 2.4	11.0 ± 7.1	0.35
CSF leak	6	0	0.19	1	0	0.4
Revision surgery	5	0	0.32	0	0	N/A

## Discussion

Endoscopic spine surgery is gaining popularity as an ultra-minimally invasive technique for spinal decompression [17,18]. It has been shown to significantly improve patients' clinical symptomatology while reducing intraoperative tissue manipulation, postoperative back pain, and hospital stay [10-14]. Performed via interlaminar or transforaminal approaches [26], endoscopic surgery can be used for an expanding set of indications, including degenerative stenosis [20], disc herniation [14,19], and fusion cases [27]. Although technological advances have increased adoption, the equipment still requires significant technical skills to master endoscopic spine surgery. To characterize this learning curve, we explored a spine surgeon's experience integrating interlaminar and transforaminal endoscopy at a tertiary hospital. Our study demonstrated a moderate learning curve for both approaches, characterized by reductions in operative time and resource utilization with experience. While there was a greater cutoff time to familiarity in the transforaminal approach compared to interlaminar cases, surgical outcomes did not differ significantly.

Baseline characteristics differed between interlaminar and transforaminal endoscopy in terms of surgical indication. The interlaminar approach was primarily used for lumbar stenosis with neurogenic claudication, while the transforaminal approach was used for disc herniation. The efficacy of interlaminar decompression as a definitive treatment for central and lateral recess stenosis is well-documented in the literature, while transforaminal discectomies have shown direct disc access via the intervertebral foramen [28-30]. In our study, transforaminal cases were also taken to relieve cord compression in the thoracic spine. Supporting the literature, we found that both approaches exhibited strong efficacy as measured by improved patient-reported pain via the VAS.

Intraoperatively, transforaminal procedures had significantly greater X-ray utilization and supply costs per level. While not statistically significant, there was also a greater average operative time/level in the transforaminal group compared to the interlaminar cases. This supports prior studies that have exhibited more complicated trajectory planning and access difficulty, which collectively require additional time and fluoroscopic guidance [31]. The docking method for the interlaminar approach is also similar to the microscopic tubular approach, while the transforaminal docking is unfamiliar to most spine surgeons. The Jamshidi needle also appeared to be a significant factor in increasing the supply cost for the transforaminal approach compared to the interlaminar. Subtype analyses in the broader literature have attributed this discrepancy in operative times to the narrow surgical window and the technical complexity of transforaminal approaches [31].

Despite differences in surgical technique, both interlaminar and transforaminal endoscopy exhibit significant learning curves as cited in the literature. Lee et al. reviewed 223 patients with lumbar canal or lateral recess who underwent endoscopic decompression by a single surgeon via laminectomy or foraminotomy [30]. They found that operative time decreased with case number and plateaued around 100 cases. Notably, results were reported in aggregate as opposed to by approach, and the study highlighted the significant endoscopic experience of the surgeon as opposed to our focus on early-stage learning curves. Nomura et al. reported a single surgeon's experience with microendoscopic laminectomies in 480 patients with lumbar canal stenosis and found a steep decline in operative time with case number but did not report a time to proficiency given the surgeon's prior expertise with endoscopic procedures [32]. However, intraoperative complications such as blood loss appeared to stabilize after 30 cases. Similarly, Son et al. found a significant decline in operative time among 48 patients who underwent transforaminal discectomies for lumbar disc herniation [33]. Regarding other aspects of surgeon familiarity, literature from various surgical domains has explored the relationship between experience and total supply cost. For example, a study of urologists by Hampson et al. found that a higher volume of approach-specific cases was a significant predictor of lower supply costs [34].

Expanding from the prior literature, we explored approach-specific analyses and comprehensively evaluated the early-stage learning curve using trends in operative time, X-ray usage, and supply cost. With increased experience, there was a decreasing trend in operative time/level and X-ray utilization/level in both approaches. While significance was found in X-ray images/level in the interlaminar approach, additional evaluation with a larger sample size may uncover further significance. Supply-cost/level had a positive relationship in both the interlaminar and transforaminal approaches, reflecting either surgeon-specific or approach-specific consistency with supply requirements. Overall, our findings reflect the surgeon's increasing efficiency and comfort with full-endoscopic surgery as experience grew.

While linear regression analysis can indicate the relationship between surgical metrics over time, cumulative running analyses have been sparingly cited to determine a fixed cutoff point for familiarity [33,35,36]. In lumbar transforaminal discectomies, Son et al. found a cutoff of 25 cases (out of 48) with a significantly decreased average operative time in the post-cutoff group (n = 23) [33]. Compared to transforaminal approaches, Park et al. reported a later competency cutoff of 58 cases in biportal endoscopic laminectomies [36]. Unlike Son et al., the present study predefined success as an operation time (< 75 minutes) and thus employed a binary cumulative sum analysis (under or over 75 minutes) to determine their cutoff point [33]. In the broader literature, however, there is no clear relationship between the surgical approach and time to competency.

To provide a multidimensional view of competency, we employed a cumulative running median analysis not only of operative time but also of X-ray usage and supply cost. Cutoff points in the interlaminar group were generally earlier than those in the transforaminal approaches. The presence of cutoffs in the first week with operative time (0.5 weeks) and cost (0.25 weeks) suggests greater surgeon familiarity with the interlaminar approach. Earlier case-based cutoffs, which included X-ray usage (nine cases vs. 11) and cost (two cases vs. 11), may have also been influenced by greater experience with interlaminar approaches, as these cases were performed more frequently. With respect to case-based competency with operative time, both interlaminar cases and transforaminal cases exhibited operative time cutoffs at three cases. These findings demonstrate significant learning occurring in both approaches, even early on in technique adoption, and an overall shortened learning curve with more frequent exposure to a given approach. It is important to note that these results may be impacted by the relatively fewer number of transforaminal cases in this study, which may have resulted in a local cutoff time in the cumulative average plot.

An additional important aspect of this learning curve is that these cases occurred in a tertiary academic hospital. A multicenter study by Phan et al. evaluated surgical times in spine surgeries and found significantly longer surgical durations when surgical trainees were involved [37]. This included procedures with medium and long-predicted durations as well as cervical and lumbar surgeries. Looking at endoscopic cases specifically, the effect of the practice setting on learning curves was further exemplified in Morgenstern et al. [23] and Ransom et al. [22]. These studies reported single-surgeon experiences with endoscopy adoption in the private practice setting using the MacNab outcomes criteria ("excellent/good," "fair," and "poor"). Surgeon competency was attained in 15 cases and 72 cases in Ransom et al. and Morgenstern et al., respectively [22,23]. Regarding the latter cutoff in the latter study, relatively stricter criteria were utilized for competency (i.e., 90% "excellent/good" outcomes). Given the results of these studies, our reported learning curves may be impacted by the professional demographics of the surgical team as well as the learning experience of various surgical trainees. At the same time, early resident exposure may significantly reduce the systemic learning curve and increase widespread adoption in the long term.

Ultimately, while markers of technical familiarity improved with experience, surgical outcomes were consistently favorable. EBL/level was similar pre- and post-cutoff time, and rates of durotomy and revision surgery were minimal. Of those who had a durotomy, none required lumbar drainage nor developed a symptomatic CSF leak. Disposition was also similar between the two approaches, with most patients being discharged home.

Limitations

This study is limited by its retrospective analysis of a single surgeon's experience, but it accurately demonstrates the challenges associated with incorporating endoscopy in a complex academic center. While this may limit the statistical power of our study and impact generalizability, we account for this by providing a head-to-head comparison of each approach's first 13-15 cases to provide size-matched results. We hope that this work will inspire further research to determine the generalizability of our findings.

## Conclusions

Our findings reaffirm that endoscopic spine surgery, along with being an effective approach to decompression, can be conducted safely throughout the moderate learning and supply cost curves. In this single-surgeon experience, significant learning occurred even at the early stage of endoscopic adoption, with overall time to familiarity expedited by more frequent exposure. While a surgeon's learning curve may be influenced by their site of practice (private practice vs. academic settings), greater exposure to endoscopic techniques during surgical training years may significantly reduce the time to competency in the future.
